# Molecular Profiling of the *Drosophila* Antenna Reveals Conserved Genes Underlying Olfaction in Insects

**DOI:** 10.1534/g3.119.400669

**Published:** 2019-09-16

**Authors:** Pratyajit Mohapatra, Karen Menuz

**Affiliations:** * Physiology & Neurobiology Department,; † Institute for Systems Genomics, and; ‡ Connecticut Institute for the Brain and Cognitive Sciences, University of Connecticut, Mansfield, CT 06269

**Keywords:** Gene Expression, Drosophila, Insect, Olfaction, Antenna

## Abstract

Repellent odors are widely used to prevent insect-borne diseases, making it imperative to identify the conserved molecular underpinnings of their olfactory systems. Currently, little is known about the molecules supporting odor signaling beyond the odor receptors themselves. Most known molecules function in one of two classes of olfactory sensilla, single-walled or double-walled, which have differing morphology and odor response profiles. Here, we took two approaches to discover novel genes that contribute to insect olfaction in the periphery. We transcriptionally profiled *Drosophila melanogaster* amos mutants that lack trichoid and basiconic sensilla, the single-walled sensilla in this species. This revealed 187 genes whose expression is enriched in these sensilla, including pickpocket ion channels and neuromodulator GPCRs that could mediate signaling pathways unique to single-walled sensilla. For our second approach, we computationally identified 141 antennal-enriched (AE) genes that are more than ten times as abundant in *D. melanogaster* antennae as in other tissues or whole-body extracts, and are thus likely to play a role in olfaction. We identified unambiguous orthologs of AE genes in the genomes of four distantly related insect species, and most identified orthologs were expressed in the antenna of these species. Further analysis revealed that nearly half of the 141 AE genes are localized specifically to either single or double-walled sensilla. Functional annotation suggests the AE genes include signaling molecules and enzymes that could be involved in odorant degradation. Together, these two resources provide a foundation for future studies investigating conserved mechanisms of odor signaling.

Vector borne diseases sicken hundreds of millions of people worldwide each year. Most infections are contracted through the bites of blood-feeding arthropods (World Health Organization 2017). The principal cues that disease vectors use to locate humans are kairomones, odors released from human sweat, skin, and breath (Takken and Knols 2010; Ray 2015). Many of the most effective means to reduce disease burden prevent vectors from contacting hosts through manipulation of their olfactory driven behaviors (Takken and Knols 2010).

The general anatomy of insect olfactory systems is conserved. Odors are detected by odor receptors located on the dendrites of olfactory receptor neurons (ORNs). A small number of ORNs and non-neuronal auxiliary cells are housed within sensory hairs known as sensilla, and hundreds of these sensilla densely cover the primary insect olfactory organ, the antenna (Shanbhag *et al.* 1999). There are two major morphological classes of sensilla, single-walled and double-walled (Keil 1999). In most organisms, pheromones and food and plant odors are detected by single-walled sensilla (Steinbrecht 1997). Many microbial products, such as acids and amines, are detected by double-walled sensilla (Steinbrecht 1997). In the widely studied olfactory system of *Drosophila melanogaster*, basiconics and trichoids are types of single-walled sensilla and coeloconics are double-walled sensilla (Shanbhag *et al.* 1999).

Insects utilize a large number of receptors to detect and discriminate the immense number and variety of odors that exist in the environment. Research over the past two decades has characterized two large odor receptor families: Ionotropic Receptors (IRs) and Odorant Receptors (ORs) (Joseph and Carlson 2015; Robertson 2019). In addition to odor receptor co-receptors, most ORNs express only one or a few members of one receptor family and these largely determine the ORN’s odor response profile (Couto *et al.* 2005; Fishilevich and Vosshall 2005; Benton *et al.* 2009; Joseph and Carlson 2015). In *Drosophila melanogaster*, OR receptors are expressed by ORNs in single-walled sensilla, and IR are receptors expressed in double-walled sensilla (Joseph and Carlson 2015). Recent studies suggest this segregation also holds in other insect species (Pitts *et al.* 2004; Yang *et al.* 2012; Guo *et al.* 2014).

Numerous studies have examined the contribution of individual odor receptors to the detection of particular odorants. Such studies have been primarily carried out in *Drosophila* due to the number of available genetic tools that support molecular manipulations of specific receptors and neurons (Hallem and Carlson 2006). More recently, genomic studies and antennal transcriptome analysis have led to identification of odor receptors in non-model organisms, including mosquitoes and other vectors of disease (Grosse-Wilde *et al.* 2011; Liu *et al.* 2012; Rinker *et al.* 2013; Hansen *et al.* 2014; Matthews *et al.* 2016). With the exception of odor receptor co-receptors, most odor receptors are poorly conserved across species (Croset *et al.* 2010; Hansson and Stensmyr 2011; Andersson *et al.* 2015), making them poor targets for broad-spectrum insect repellents. This lack of conservation is consistent with the consensus that odor receptors evolve rapidly to reflect the ecology of particular organisms (Carey *et al.* 2010; Robertson 2019).

Currently there is a surprising lack of knowledge regarding specific molecules other than odor receptors that play critical roles in mediating the first steps of odor detection in the antenna. Such molecules could underlie signal amplification, adaptation, odor degradation, signal termination, the transepithelial electrical potential, or other signaling processes (Stengl *et al.* 1999; Leal 2013; Guo and Smith 2017). Importantly, such genes may show broader conservation across species than the odor receptors themselves.

Genes with a role in insect olfaction have been sought previously using behavioral screens to identify smell impaired mutants (McKenna *et al.* 1989; Anholt *et al.* 1996; Arya *et al.* 2015). These screens could not distinguish genes that play a role in olfactory circuits in the brain from those that contribute to odor responses in the antenna. More recent work has characterized gene expression in the antenna using RNA-Seq transcriptional profiling in *Drosophila* and other species (Grosse-Wilde *et al.* 2011; Liu *et al.* 2012; Rinker *et al.* 2013; Hansen *et al.* 2014; Menuz *et al.* 2014; Younus *et al.* 2014; Matthews *et al.* 2016). The challenge lies in identifying those genes that play a specific role in olfaction rather than mediating functions that generally support neuronal or antennal physiology. Genes that play critical roles in olfaction are not necessarily those with the highest antennal expression; for example, most odor receptors are expressed at relatively low levels (<50 FPKM) (Shiao *et al.* 2013; Menuz *et al.* 2014). One successful approach to overcoming this challenge is transcriptional profiling of genetic mutants. By comparing antennal gene expression in wild-type flies and *atonal* mutants that lack double-walled sensilla, we previously identified an uncharacterized transporter with an unexpectedly critical role in maintaining ORN activity (Menuz *et al.* 2014).

Here, we took two approaches to identify genes that are likely to play a role in antennal olfactory signaling. We first identified genes that are enriched in single-walled sensilla by generating an antennal transcriptome dataset for *amos* flies, which selectively fail to develop these sensilla (zur Lage *et al.* 2003). This dataset revealed 187 *amos*-depleted genes, including 35 expected members of the OR family. We then took a computational approach to identify 141 genes whose expression is greatly enriched in the *Drosophila* antenna. Orthologs of the antennal-enriched genes were identified in four insect species separated by >300 million years of evolution, and most orthologs were expressed in antennal transcriptomes of their respective species. Half of the AE genes could be mapped to either single or double walled sensilla, and functional annotation revealed new candidates for mediating signal amplification, adaptation, and odor degradation. Together, our two approaches have identified dozens of novel candidate olfaction genes that will be a boon for future studies of olfactory signaling in the periphery.

## Materials and Methods

### Drosophila melanogaster stocks

Mutant *amos*^*1*^* pr^1^*/Cyo flies (zur Lage *et al.* 2003) were obtained from Richard Benton, and the line was outcrossed for ten generations to Canton-S wild-type flies to remove a lethal mutation. These outcrossed homozygous *amos* mutant flies and Canton-S wild-type flies were used for RNA-Seq studies. Canton-S flies were used for qRT-PCR experiments.

### Total RNA extraction from fly tissues

Canton-S and *amos* flies (3-5 days old) were frozen in liquid nitrogen, and tissues were dissected into 1.5 ml Eppendorf tubes kept in liquid nitrogen. For RNA sequencing, each sample consisted of 300-400 antennae from approximately equal numbers of males and females. Analysis of the expression of known auditory organ genes found in the second antennal segment indicates that our samples predominantly contained antennal third (olfactory) segments as intended. Many of the most highly expressed genes in the second (auditory) segment of the antennae are not expressed in our CS dataset (File S1, “auditory gene expression”) (Senthilan *et al.*, 2012).

For quantitative reverse transcriptase PCR (qRT-PCR), tissue samples consisted of legs (∼50), antennae (∼200), entire bodies without heads and legs (∼10), and heads (∼10). Tissues were ground using disposable RNAse-free pestles and a QIAshredder column (Qiagen). Total RNA was extracted using RNeasy Micro Kits (Qiagen) for qRT-PCR and an RNeasy Mini Kit (Qiagen) for RNA-Seq. For both, the manufacturer’s RNeasy Mini Kit protocol was followed as it gave better yield and purity. To increase the yield, the RNeasy spin column was rolled horizontally during the RW1 and RPE steps to ensure no sample remained on the wall and cap of the RNeasy spin column, and RLT was gently warmed before use.

### Next generation sequencing

Three independent antennal RNA samples were collected from Canton-S and *amos* flies and then cleared of genomic DNA using the DNAse in an iScript gDNA Clear cDNA Synthesis Kit (Bio-Rad). The six cleared RNA samples (∼0.5 µg each) were sent to the Center for Genome Innovation at the University of Connecticut for quality control, library preparation, and NextGen sequencing. Libraries were prepared using the Illumina mRNA sample prep kit for non-stranded RNA, and sequencing was carried out on an Illumina NextSeq 500 with 150 cycles to produce paired-end reads.

### RNA-Seq quantification and differential expression analysis

Raw reads were downloaded from BaseSpace Sequence Hub (Illumina). Quality control on the reads was performed using Sickle with the default parameters to produce trimmed reads (Joshi 2011). The reference *Drosophila melanogaster* genome and gene annotation file were downloaded from NCBI (dm6, 2014, Release 6 plus ISO1 MT) (dos Santos *et al.* 2014). STAR was used with the default parameters to align trimmed reads to the reference genome with mitochondrial genes removed (Dobin *et al.* 2013). Programs were executed using the University of Connecticut High Performance Computing cluster. All mapped reads generated by STAR have been submitted to the NCBI SRA and are available under the BioProject accession number PRJNA532415.

Two methods were used to call gene expression. For differential expression analysis comparing CS and *amos* flies, HTSeq was used to obtain raw counts of reads mapping uniquely to each gene (Anders *et al.* 2015). HTSeq-count was used with the “intersection-nonempty” and “nonunique-none” modes to handle reads mapping to more than one feature (File S1, “HTSeq”). Raw counts were then analyzed using EdgeR v3.8 package in R studio v3.4.2 to identify differential gene expression (Robinson *et al.* 2010) (File S1, “*amos* EdgeR analysis”); this was the same method as used previously for *atonal* analysis (Menuz *et al.* 2014). Genes with a FDR <0.01 and more than fourfold change in expression between *amos* and CS flies were considered differentially expressed. Of these 341 genes, 187 were reduced in *amos* flies.

Using analogous criteria, we also identified 154 genes that were upregulated in *amos* mutants (File S1, “*amos* EdgeR analysis”). Closer analysis revealed that for 68 of these genes, all wt samples and two *amos* samples had very low expression (average RPM 1.8), whereas there was some increased expression in *amos* sample 3 likely due to slight contamination by an additional tissue. Therefore, we considered the remaining 86 genes to be those that were truly upregulated in *amos* mutants (File S1, “*amos* upregulated-genes”). To be certain that this variability in *amos* sample 3 did not influence our identification of *amos*-depleted genes, we verified that the expression values of each of the 187 *amos*-depleted genes were similar in all three samples of a particular genotype.

We used Cufflinks to compare gene expression across tissues because this was similar to the data processing used for the ModEncode datasets (Graveley *et al.* 2011; Trapnell *et al.* 2012; Brown *et al.* 2014) and because Cufflinks could provide values for gene expression as fragments per kilobase per million-mapped reads (FPKM). Cufflinks handles ambiguous reads by estimating transcript abundances, considering biases such as non-uniform distribution along the gene length. Library type was selected as “fr-firststrand” and the maximum number of fragments per locus was set to 1 billion in order to include genes with very high number of reads mapped to them, such as some OBPs. All other parameters were set to default. Per sample, 84–92% of total reads were aligned, for a total of ∼4.3 to 7.6 million aligned reads per sample (File S1, “Cufflinks”).

### Identifying antennal enriched genes in Drosophila

There were 4,129 genes in CS wild-type antennae that are expressed >1 FPKM in each of the three samples and with an average expression of at least 10 FPKM. Expression levels of these genes in other tissues were obtained from datasets generated by the modENCODE consortium and downloaded from FlyBase (Roy *et al.* 2010; Graveley *et al.* 2011; Brown *et al.* 2014; Thurmond *et al.* 2019). These included transcriptomes of different *Drosophila melanogaster* tissues (larval digestive system, 4 days old adult digestive system, larval fat body, pupal fat body, larval salivary gland, pupal salivary gland, larval central nervous system, pupal central nervous system, 4 days old adult male head, and 4 days old adult female head) and whole bodies (embryo 22-24 hr, larva L1, larva L3, pupa P5, pupa P15, 5 days old adult male and 5 days old adult female). Custom scripts written in Spyder3.2.3, a scientific Python development environment from the Anaconda navigator, were used to create two tables: 1) the 4,129 antennal genes, their antennal expression, and their expression in other tissues, and 2) the 4,129 antennal genes, their antennal expression, and their expression in whole bodies over development. Genes with >10-fold higher expression in antennae compared to the ten tissue datasets and genes with >10-fold higher expression in antennae compared to seven whole body developmental time points were identified. The two gene datasets were then merged to identify 177 genes enriched in both. Automatic annotation using FlyBase determined that 141 of these genes encoded proteins, and these were considered the set of coding antennal-enriched (AE) genes for further analysis (File S1, “antennal-enriched genes”).

### Quantitative real-time PCR validation of AE genes

Seven of the AE genes that had not been studied previously were selected for validation with qRT-PCR experiments. Equal amounts of RNA extracted from dissected fly tissues (200 ng per sample) were used to generate cDNA using the iScript gDNA Clear cDNA Synthesis Kit (Bio-Rad). The cDNA was used as a template for qRT-PCR with the SsoFast EvaGreen Supermix (Bio-Rad), similar to (Menuz *et al.* 2014). The housekeeping gene *eIF1A* was used to normalize total cDNA levels between tissues. A CFX96 thermocycler (Bio-Rad) was used for the qRT-PCR assays. Gene expression in each tissue was measured relative to the expression of *eIF1A* in that sample. Each reaction was run on three to five independent tissue replicates. Differences between samples were analyzed with ANOVA followed by the Dunnett’s *post hoc* test using GraphPad Prism 7.

The following primer pairs were used:

*eIF1A*: ATCAGCTCCGAGGATGACGC and GCCGAGACAGACGTTCCAGA

*CG10357*: GCAGTCATGTTCTACGCTG and CCTTAGGCAGTCCTCTACA

*CG14142*: GCACTTAACATGCCTAAAAGT and CGAAAGTATTCACCGCCGA

*CG34309*: CCATTTGCGGATTTGTACTTC and ATTCGTTCTTCAGTCAGCGG

*CG42284*: CCAGAGTCAGAGAACGATAACG and CCATTGCTGATGTGAGTGAC

*CG14661*: CCTGTTGATCTGTTTTGTAGC and CAGTTCCTTAATGCCCTTGG

*CG9541*: CCAGTCGCCGATTATTTCG and GTATCCCTCTGCCCATACC

*CG14445*: GTGCGAAAAGAACGAGTTCC and CGTAGGATGGGGTCAATAGG

### Analysis of ortholog expression in the antennae of other insect species

We searched for orthologs in other insect species for 96 of the AE genes. Known chemoreceptors (IRs/ORs/Grs) and OBPs were excluded because their conservation has been extensively researched in prior studies. The UniProt ID for each of the 96 genes was obtained using the Flybase gene symbol as a query in UniProtKB (UniProt Consortium 2018). Orthologs of these proteins were identified using InParanoid 8 using the *Drosophila* AE gene UniProt ID as the query (Sonnhammer and Östlund 2014). Four insect species were selected: *Harpegnathos saltator* (ants), *Tribolium castaneum* (beetles), *Anopheles gambiae* (mosquitos), and *Apis mellifera* (bees). These were selected because they represent a wide distribution along the insect phylogenetic tree, and because antennal transcriptomes for these species could be found in the NCBI SRA (Leinonen *et al.* 2010; Missbach *et al.* 2014). Conservative criteria were applied to identify unambiguous orthologs and to exclude inparalogs. Orthologs were identified when the bootstrap value for the match was >90% and the identified ortholog mapped back to the original *Drosophila* gene when used in a BLAST search of *Drosophila melanogaster* proteins.

The NCBI SRA was utilized to determine if the AE gene orthologs are expressed in the antennae of their respective insect species. To do this, the UniProt gene IDs of the orthologs were first used to obtain the corresponding RefSeq mRNA sequences. These mRNA sequences were used to query the antennal transcriptomes of the respective species using NCBI SRA-BLAST (*Harpegnathos saltator*, SRX1164271; *Tribolium castaneum*, SRX757109; *Apis mellifera*, SRX518058; *Anopheles gambiae*, SRX765675) (Zhou *et al.* 2012; Dippel *et al.* 2014; Hodges *et al.* 2014; Jasper *et al.* 2014). We later interrogated two additional transcriptomes for Figure S1 using a second *Tribolium castaneum* dataset, SRX757107, and one from *Bombyx mori*, SRX3181880 (Dippel *et al.* 2014; Qiu *et al.* 2018). Perfect matches (32-100 bp depending on the deposited read lengths) were used to identify expression of each ortholog. The number of reads mapping to a gene was used to estimate its antennal expression in FPKM based on the mRNA length and total number of reads in the sample. Genes were considered expressed in the antenna if they had expression >3 FPKM. To analyze expression of random genes in the antennal transcriptomes, we randomly selected genes from *Drosophila*, identified orthologs in the other insect genomes, and determined the percentage of orthologs expressed in the antennal transcriptomes, similar to the analysis of the AE gene orthologs.

We note that after completing the study, we learned that the original paper for the *Anopheles* transcriptome was retracted due to contamination of samples from the maxillary palp, but not those from the antenna (Hodges *et al.* 2015). Therefore, our analysis of the antennal dataset should not be affected.

### Functional annotation of amos-depleted and AE genes

FlyBase, NCBI BLAST, and DAVID were used to manually curate protein sequences (Huang *et al.* 2009; Thurmond *et al.* 2019). FlyBase integrates information from different databases including GO terms, UniProt protein families, and InterPro protein domains. SignalP 4.1 and TMHMM v.2.0 were used to identify signal peptide sequences and transmembrane domains and DeepLoc to infer cellular localization (Möller *et al.* 2001; Almagro Armenteros *et al.* 2017; Nielsen 2017). Significant enrichment of GO terms in the *amos*-depleted dataset was determined using PANTHER 14.0 in AmiGO 2 with GO database release 2018-12-01 (Ashburner *et al.* 2000; Carbon *et al.* 2009; Mi *et al.* 2016; Gene Ontology Consortium 2019).

### Data availability

All raw read data are available at BioProject accession number PRJNA532415. Analyzed data reporting gene expression are available in File S1. Supplemental material available at FigShare: https://doi.org/10.25387/g3.9820682.

## Results

### Identification of genes highly expressed in single-walled sensilla

Given the morphological and molecular differences of the single and double-walled sensilla, it is likely that they have unique molecular signaling pathways. The function of single-walled sensilla has been of particular interest due to their importance in the detection of pheromones by many insects and host-derived odors by insect vectors of disease (Kaissling *et al.* 1989; Degennaro *et al.* 2013; Mcmeniman *et al.* 2014; Ghaninia *et al.* 2018; Chahda *et al.* 2019). Previously we successfully used transcriptional profiling of *atonal* antennae, which lack double-walled sensilla, to identify a novel gene essential for the response of ORNs to ammonia (Menuz *et al.* 2014). We therefore decided to take a similar genetic approach to identify genes enriched in *Drosophila* single-walled sensilla.

**Figure 1:**
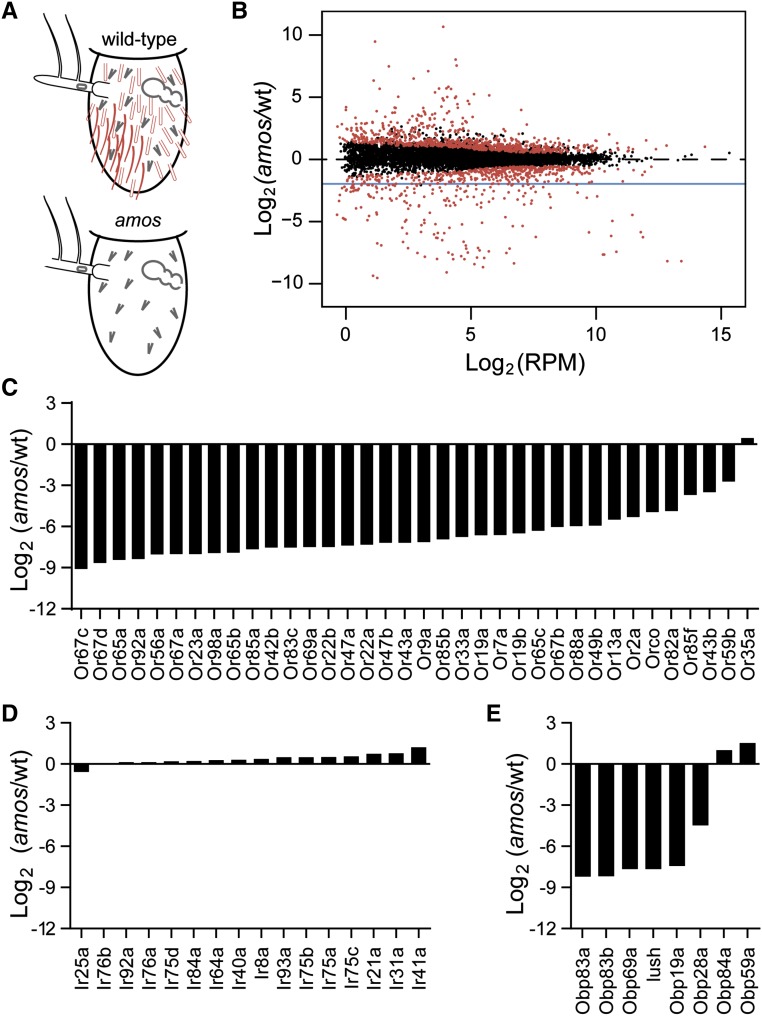
Identification of genes selectively expressed in single-walled sensilla. (A) *amos* flies fail to develop single-walled sensilla, including trichoid (red filled) and basiconic (red outline) subtypes in contrast to wild-type flies, whereas coeloconic sensilla are present in normal numbers (gray). Schematic based on findings from zur Lage *et al.*, 2003 and adapted from Menuz *et al.*, 2014. (B) Scatterplot showing the *Drosophila* genes (dots) plotted based on their average expression level in RPM and the expression ratio between *amos* and wt flies. Genes were considered *amos*-depleted if they were differentially expressed with FDR <0.01 (red) and were at least fourfold reduced in *amos* flies (below blue line). (C) Expression of antennal OR genes is greatly reduced in *amos* mutants, but (D) IR expression is not. (E) Six OBPs found in single-walled sensilla (*Obp83a*, *Obp83b*, *Obp69a*, *lush*, *Obp19a*, and *Obp28a*) have greatly reduced expression in *amos* antennae, but two Obps found in double-walled sensilla (*Obp84a* and *Obp59a*) do not.

To do this, we used RNA-Seq to compare antennal expression of genes in wild-type and *amos* antennae, which lack single-walled sensilla including basiconic and trichoid sensilla, but have normal numbers of double-walled sensilla (Figure 1A) (zur Lage *et al.* 2003). These flies entirely lack single-walled sensilla cells, including ORNs and support cells that derive from sensory organ precursors (SOPs) in development (zur Lage *et al.* 2003). We first outcrossed the *amos* line for ten generations to wild-type (wt) Canton-S flies to remove a nearby lethal mutation. We then carried out next generation transcriptional profiling on three sets of antennae from *amos* and wt flies (File S1, “HtSeq”). As expected, most genes are expressed at similar levels in the two genotypes (Figure 1B). This is consistent with the idea that many genes have a ubiquitous role across the sensillar populations. However, the expression of 187 genes is statistically reduced at least fourfold in *amos* mutants (File S1, “amos edgeR analysis”). We considered these genes *amos*-depleted.

We then examined the expression of genes known to be localized to single-walled sensilla to validate the specificity of the *amos* mutation and our approach. Our wt dataset contained 36 members of the OR receptor family that were expressed in the wild-type antenna, and all but one gene was *amos*-depleted (Figure 1C). The exception, *Or35a*, is the only member of the OR family expressed in double-walled sensilla (Couto *et al.* 2005). Conversely, none of the 16 IR family members were identified as *amos*-depleted (Figure 1D). We also examined the predicted localization of eight antennal Odorant Binding Proteins (OBPs) that were detected by *in situ* hybridization in either single or double-walled sensilla (Larter *et al.* 2016). The six OBPs detected in single-walled sensilla were *amos*-depleted, whereas the two OBPs found in double-walled sensilla were not (Figure 1E). These data confirm that single-walled sensilla are indeed selectively lost in *amos* antennae and that our approach identifies genes enriched in single-walled sensilla. We note that although most of these 187 genes are likely enriched in single-walled sensilla, there could be some remaining genetic background differences between *amos* and wt flies after outcrossing, and this could contribute to some of the observed differences in expression for some genes.

### Many of the single-wall sensilla enriched genes are transmembrane proteins

**Figure 2:**
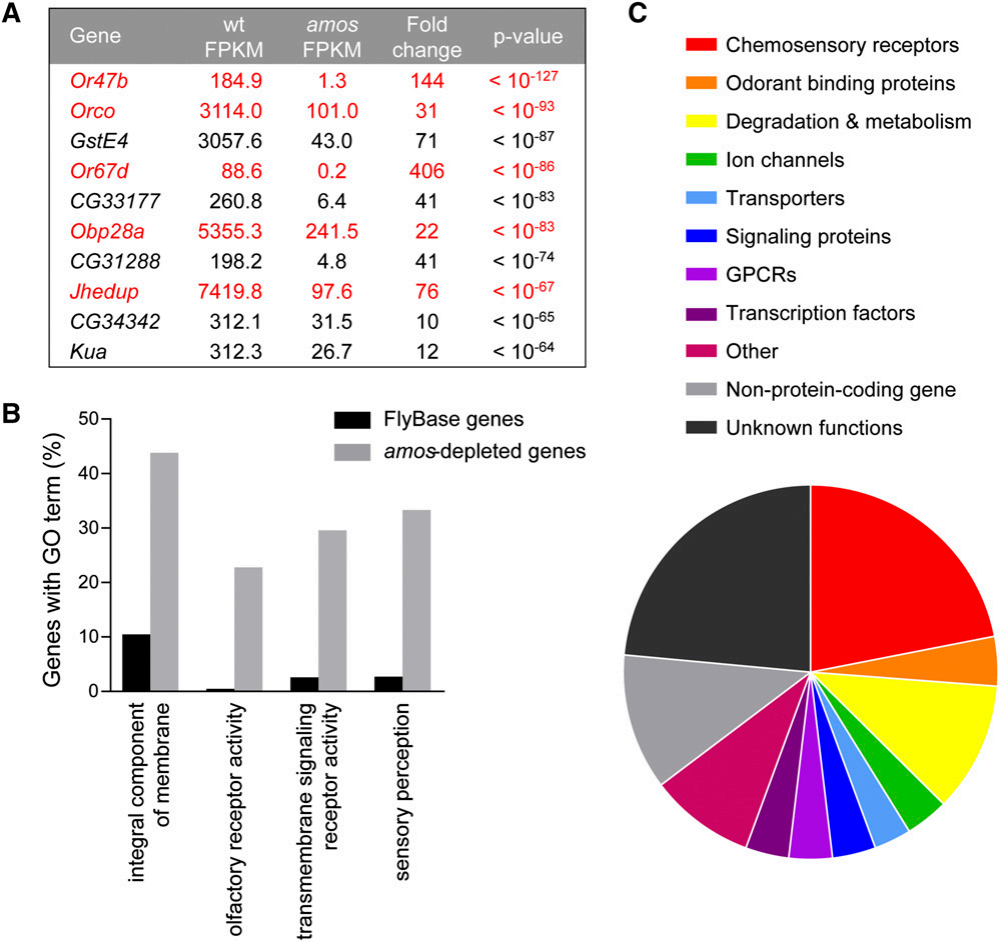
*amos*-depleted genes fulfill a variety of molecular functions. (A) The top ten differentially expressed genes based on p-value include five genes with known localization to single-walled sensilla (red). (B) GO term enrichment analysis of the 187 *amos*-depleted genes compared to all *Drosophila* genes listed in FlyBase reveals several enriched GO terms. (C) The chart depicts the frequency of *amos*-depleted genes falling into different functional categories.

Having validated the use of *amos* mutants, we next looked more closely at the 187 genes identified as *amos*-depleted (File S1, “amos edgeR analysis”). The ten genes reduced with the greatest statistical significance included five genes previously shown to be localized to single-walled sensilla: three members of the OR family, *Obp28a*, and an enzyme *Jhedup* (Figure 2A) (Couto *et al.* 2005; Larter *et al.* 2016; Steiner *et al.* 2017). The localization of the other four genes had not been studied in the antenna previously.

We then turned to Gene Ontology annotation to determine if particular categories of genes were enriched among the *amos*-depleted genes. Using AmiGO 2, we found that 162 of 187 *amos*-depleted genes had been previously annotated with Gene Ontology (GO) terms (Ashburner *et al.* 2000; Carbon *et al.* 2009; Gene Ontology Consortium 2019). The frequency of GO terms in our dataset was statistically compared to their frequency among all FlyBase genes using PANTHER version 14 (Figure 2B) (Mi *et al.* 2016). This revealed that the *amos*-depleted genes are particularly enriched in transmembrane proteins; the GO term “integral component of membrane” was associated with 43.8% of *amos*-depleted genes *vs.* 10.5% of FlyBase genes (*P* < 10^−23^). This is in part due to the enrichment of genes labeled with the molecular functions “olfactory receptor activity” (22.8% in *amos*-depleted *vs.* 0.5% of FlyBase genes, *P* < 10^−41^) and “transmembrane signaling receptor activity” (29.6% in *amos*-depleted *vs.* 2.6% of FlyBase genes, *P* < 10^−31^). Genes associated with the GO biological process term “sensory perception” were also highly enriched among the *amos*-depleted genes (33.3%) compared to 2.8% of FlyBase genes (*P* < 10^−37^).

Functional annotation of the *amos*-depleted genes was accomplished with BLAST searches and FlyBase tools (Figure 2C, Table 1) (Thurmond *et al.* 2019). Putative functions could be assigned to ∼75% of the genes. In addition to the large number of OR receptors, our dataset included six members of the GR gustatory receptor family (*Gr21a*, *Gr43a*, *Gr63a*, *Gr64a*, *Gr64f*, and *Gr93a*). Together OR and GR chemoreceptors represent ∼20% of the *amos*-depleted genes.

**Table 1:** 187 genes depleted in *amos* antennae

FBgn ID	Symbol	Function
**CHEMOSENSORY RECEPTORS**		
FBgn0030715	Or13a	OR chemosensory receptor
FBgn0041626	Or19a	OR chemosensory receptor
FBgn0062565	Or19b	OR chemosensory receptor
FBgn0026398	Or22a	OR chemosensory receptor
FBgn0026397	Or22b	OR chemosensory receptor
FBgn0026395	Or23a	OR chemosensory receptor
FBgn0023523	Or2a	OR chemosensory receptor
FBgn0026392	Or33a	OR chemosensory receptor
FBgn0033043	Or42b	OR chemosensory receptor
FBgn0026389	Or43a	OR chemosensory receptor
FBgn0026393	Or43b	OR chemosensory receptor
FBgn0026386	Or47a	OR chemosensory receptor
FBgn0026385	Or47b	OR chemosensory receptor
FBgn0028963	Or49b	OR chemosensory receptor
FBgn0034473	Or56a	OR chemosensory receptor
FBgn0034865	Or59b	OR chemosensory receptor
FBgn0041625	Or65a	OR chemosensory receptor
FBgn0041624	Or65b	OR chemosensory receptor
FBgn0041623	Or65c	OR chemosensory receptor
FBgn0036009	Or67a	OR chemosensory receptor
FBgn0036019	Or67b	OR chemosensory receptor
FBgn0036078	Or67c	OR chemosensory receptor
FBgn0036080	Or67d	OR chemosensory receptor
FBgn0041622	Or69a	OR chemosensory receptor
FBgn0030016	Or7a	OR chemosensory receptor
FBgn0041621	Or82a	OR chemosensory receptor
FBgn0037399	Or83c	OR chemosensory receptor
FBgn0037576	Or85a	OR chemosensory receptor
FBgn0037590	Or85b	OR chemosensory receptor
FBgn0037685	Or85f	OR chemosensory receptor
FBgn0038203	Or88a	OR chemosensory receptor
FBgn0038798	Or92a	OR chemosensory receptor
FBgn0039551	Or98a	OR chemosensory receptor
FBgn0030204	Or9a	OR chemosensory receptor
FBgn0037324	Orco	OR chemosensory receptor
FBgn0041250	Gr21a	GR chemosensory receptor
FBgn0041243	Gr43a	GR chemosensory receptor
FBgn0035468	Gr63a	GR chemosensory receptor
FBgn0045479	Gr64a	GR chemosensory receptor
FBgn0052255	Gr64f	GR chemosensory receptor
FBgn0041229	Gr93a	GR chemosensory receptor
**ODORANT BINDING PROTEINS**		
FBgn0020277	lush	Odorant Binding Protein
FBgn0010403	Obp83b	Odorant Binding Protein
FBgn0011283	Obp28a	Odorant Binding Protein
FBgn0046878	Obp83cd	Odorant Binding Protein
FBgn0011281	Obp83a	Odorant Binding Protein
FBgn0031109	Obp19a	Odorant Binding Protein
FBgn0046876	Obp83ef	Odorant Binding Protein
FBgn0011279	Obp69a	Odorant Binding Protein
**TRANSPORTERS**		
FBgn0025709	CNT2	SLC28 nucleoside transporter
FBgn0033685	OSCP1	Organic solute carrier partner 1 (OSCP1)
FBgn0032879	CarT	SLC22 carcinine transporter
FBgn0039915	Gat	SLC6 GABA transporter
FBgn0038716	CG7342	SLC22 transporter
FBgn0031998	SLC5A11	SLC5 sodium/sugar transporter
**ION CHANNELS**		
FBgn0003861	trp	transient receptor potential channel
FBgn0031803	ppk14	sodium channel
FBgn0039679	ppk19	sodium channel
FBgn0053289	ppk5	sodium channel
FBgn0034489	ppk6	sodium channel
FBgn0053349	ppk25	sodium channel
FBgn0036235	CG6938	anoctamin
**G-PROTEIN COUPLED RECEPTORS**		
FBgn0087012	5-HT2A	serotonin receptor
FBgn0261929	5-HT2B	serotonin receptor
FBgn0050106	CCHa1-R	CCHamide neuropeptide receptor
FBgn0052447	CG32447	class C GPCR
FBgn0030437	hec	GPCR involved in mating
FBgn0016650	Lgr1	hormone receptor
FBgn0037546	mAChR-B	acetylcholine receptor
**SIGNALING MOLECULES**		
FBgn0010223	Galphaf	G protein
FBgn0050274	CG30274	kinase
FBgn0032083	CG9541	kinase
FBgn0037167	CG11425	lipid phosphatase
FBgn0011676	Nos	nitric oxide synthase
FBgn0250862	CG42237	phospholipase A2
FBgn0002937	ninaB	retinal isomerase
**DEGRADATION & METABOLISM**		
FBgn0031426	CG18641	carboxylic ester hydrolase
FBgn0033395	Cyp4p2	cyp450
FBgn0000473	Cyp6a2	cyp450
FBgn0038029	GstD11	glutathione S transferase
FBgn0010044	GstD8	glutathione S transferase
FBgn0063496	GstE4	glutathione S transferase
FBgn0034076	Jhedup	carboxylesterase
FBgn0000075	amd	carboxylase
FBgn0263830	CG40486	estradiol 17-beta-dehydrogenase
FBgn0069973	CG40485	estradiol 17-beta-dehydrogenase
FBgn0039131	CG12268	fatty acyl coA reductase
FBgn0085371	CG34342	fatty acyl coA reductase
FBgn0000078	Amy-d	amylase
FBgn0000079	Amy-p	amylase
FBgn0050502	fa2h	oxidoreductase
FBgn0035743	Acbp6	acyl-CoA-binding protein (ACBP)
FBgn0265268	CG18234	peptidyl-proline dioxygenase
FBgn0033521	CG12896	peroxiredoxins
FBgn0051115	CG31115	S-methyl-5-thioadenosine phosphorylase
FBgn0053177	CG33177	glutathione peroxidase
FBgn0051644	CG31644	mitochondrial cytochrome-c oxidase
**TRANSCRIPTION FACTORS**		
FBgn0002633	E(spl)m7-HLH	basic helix-loop-helix transcription factor
FBgn0034096	CG7786	basic leucine zipper transcription factor
FBgn0041105	nerfin-2	C2H2 zinc finger transcription factor
FBgn0019650	toy	homeobox transcription factor
FBgn0015561	unpg	homeobox transcription factor
FBgn0020378	Sp1	Sp1/Klf family transcription factor
FBgn0000233	btd	Sp1/Klf family transcription factor
**OTHER FUNCTIONS**		
FBgn0010385	Def	antibacterial peptide
FBgn0004781	Ccp84Ac	cuticle component
FBgn0033597	Cpr47Ea	cuticle component
FBgn0039347	CG5071	cyclophilin family peptidylprolyl isomerase
FBgn0001174	halo	kinesin-1 co-factor
FBgn0260004	Snmp1	pheromone detection/lipoprotein receptor
FBgn0004511	dy	zona pellucida domain protein family
FBgn0037433	CG17919	phosphatidylethanolamine-binding protein
FBgn0029977	hdm	DNA binding protein/repair
FBgn0035608	blanks	RNA binding protein
FBgn0035626	lin-28	RNA binding protein
FBgn0029843	Nep1	metallopeptidase
FBgn0031560	CG16713	serine endopeptidase inhibitor
FBgn0262721	CG43165	serine endopeptidase inhibitor
FBgn0013433	beat-Ia	axon guidance
FBgn0038498	beat-IIa	axon guidance
FBgn0259210	prom	photoreceptor positioning
**UNKNOWN FUNCTIONS**		
FBgn0050488	antr	cystein rich secretory protein
FBgn0014000	Hf	helical cytokine/innate immunity
FBgn0032850	Kua	transmembrane protein 189
FBgn0261534	l(2)34Fc	insect defense protein
FBgn0033855	link	secreted protein, localized near midline
FBgn0042129	OS9	olfactory specific, small secreted protein
FBgn0037427	Osi17	insect Osiris transmembrane protein
FBgn0037414	Osi7	insect Osiris transmembrane protein
FBgn0037415	Osi8	insect Osiris transmembrane protein
FBgn0033042	Tsp42A	tetraspannin four transmembrane protein
FBgn0033127	Tsp42Ef	tetraspannin four transmembrane protein
FBgn0033135	Tsp42En	tetraspannin four transmembrane protein
FBgn0038028	CG10035	
FBgn0032843	CG10730	
FBgn0039297	CG11852	
FBgn0040688	CG12483	
FBgn0030886	CG12672	
FBgn0033501	CG12911	
FBgn0037013	CG13250	
FBgn0039319	CG13659	
FBgn0031219	CG13694	
FBgn0030277	CG1394	
FBgn0032734	CG15169	
FBgn0032733	CG15170	
FBgn0039723	CG15522	
FBgn0032719	CG17321	
FBgn0036923	CG17732	
FBgn0050339	CG30339	
FBgn0050356	CG30356	
FBgn0051097	CG31097	
FBgn0051288	CG31288	
FBgn0036459	CG3349	
FBgn0085195	CG34166	
FBgn0259831	CG34309	
FBgn0260657	CG42540	
FBgn0261834	CG42766	
FBgn0262858	CG43222	
FBgn0263656	CG43647	
FBgn0264443	CG43861	
FBgn0034128	CG4409	
FBgn0039346	CG5079	
FBgn0030913	CG6123	
FBgn0039728	CG7896	
FBgn0032085	CG9555	
**NON-PROTEIN CODING GENES**		
FBgn0263453	asRNA:CR43476	antisense RNA CG43476
FBgn0264874	asRNA:CR44065	antisense RNACG10874
FBgn0266144	asRNA:CR44850	antisense RNA l(2)34Fc
FBgn0266275	asRNA:CR44960	antisense RNA CG33178
FBgn0266402	asRNA:CR45042	antisense RNA Gr64d, Gr64e
FBgn0263444	asRNA:CR43467	antisense RNA Or46a
FBgn0265168	asRNA:CR44237	antisense RNA Or88a
FBgn0050009	lncRNA:CR30009	long non-coding RNA
FBgn0263509	lncRNA:CR43498	long non-coding RNA
FBgn0264382	lncRNA:CR43834	long non-coding RNA
FBgn0264438	lncRNA:CR43856	long non-coding RNA
FBgn0264835	lncRNA:CR44043	long non-coding RNA
FBgn0265312	lncRNA:CR44285	long non-coding RNA
FBgn0265376	lncRNA:CR44317	long non-coding RNA
FBgn0265718	lncRNA:CR44525	long non-coding RNA
FBgn0265734	lncRNA:CR44541	long non-coding RNA
FBgn0266828	lncRNA:CR45290	long non-coding RNA
FBgn0266859	lncRNA:CR45320	long non-coding RNA
FBgn0267058	lncRNA:CR45502	long non-coding RNA
FBgn0267938	lncRNA:CR46218	long non-coding RNA
FBgn0267965	lncRNA:CR46245	long non-coding RNA
FBgn0263472	snoRNA:2R:9445205	small nucleolar RNA

Interestingly, many *amos*-depleted genes have potential roles in neuronal signaling (Table 1). We identified 13 transporters and ion channels, including five members of the pickpocket family of degenerin/epithelial Na^+^ channels (ENaCs). There are also seven GPCRs, including serotonin and acetylcholine receptors that are known to regulate presynaptic terminals in other neuronal systems (Hoyer *et al.* 1994; Caulfield and Birdsall 1998). Additionally, there are also several signaling molecules such as kinases that are *amos*-depleted.

Similar to genes that were enriched in double-walled coeloconic sensilla, many genes enriched in single-walled sensilla are likely to function in odor degradation and metabolism such as cytochrome p450s and reductases (Table 1) (Leal 2013; Menuz *et al.* 2014). Such genes comprise more than 10% of the *amos*-depleted genes. Most have not been studied previously, although a few such as the carboxylesterase *Jhedup* and the glutathione S transferases (GSTs) *GstD11* and *GstD8* were shown to be enriched in the antenna compared to other tissues (Younus *et al.* 2014; Steiner *et al.* 2017).

Single-walled sensilla also selectively express proteins with a variety of other functions. Some genes could play a developmental role such as axon guidance molecules from the beat family and seven transcription factors, including *CG7786* and *Sp1*. The *amos*-depleted genes also include more than 20 non-protein coding genes, the majority of which are long non-coding RNAs. Interestingly, three are antisense RNAs which target OR and GR receptors.

We also looked at the putative functions of the 86 *amos*-upregulated genes (File S1, “*amos*-upregulated genes”). This set of genes included 12 non-coding RNAs, 17 biotransformation enzymes, four GPCRs, and five chitin-related genes in addition to others with diverse predicted functions. There were also four genes implicated in immune system activity and nine genes related to serine proteases and their inhibitors, which can also be involved in defense responses. This could indicate that the *amos* mutants are infected by microbes, consistent with their reduced fitness compared to wild-type flies. Other upregulated genes may be expressed in the ectopic mechanosensory bristles found on *amos* antennae (zur Lage *et al.* 2003). Some genes may be upregulated in the remaining coeloconic sensilla to compensate for the loss of olfactory sensing mediated by single-walled sensilla, which contain more than 3/4 of all ORNs (Shanbhag *et al.* 1999). For example, such compensation could explain the upregulation of the Odorant Binding Protein *Obp57e* and the chemosensory receptor *Gr98b*.

### Identification of antennal-enriched genes in Drosophila

We then sought a different approach to identify genes that are involved specifically in insect olfaction, particularly those that play a conserved role across insect species. We reasoned that many of these molecules are likely to be highly enriched in the antenna compared to other tissues in the same insect. Recently, there has been an explosion of RNA-Seq datasets quantifying the transcriptomes of various tissues (Grosse-Wilde *et al.* 2011; Liu *et al.* 2012; Rinker *et al.* 2013; Hansen *et al.* 2014; Menuz *et al.* 2014; Younus *et al.* 2014; Matthews *et al.* 2016). The RNA-Seq technique provides a quantitative measure (“FPKM”) of gene expression that reflects the portion of transcripts in a given transcriptome from that particular gene. A key advantage of RNA-Seq compared to earlier microarray technology is its quantitative nature, allowing expression of a given gene to be compared across tissues and datasets (Wang *et al.* 2009).

In order to identify conserved antennal-enriched genes, we first focused on *Drosophila melanogaster* because we could leverage the high-quality transcriptome datasets generated by the ModEncode consortium for this species (Graveley *et al.* 2011; Brown *et al.* 2014). The ModEncode consortium has systematically characterized gene expression in *Drosophila* at a high sequencing depth across several tissues and whole-body extracts at multiple developmental time points. Such depth is critical for accurate quantification of gene expression in tissues with low expression.

**Figure 3:**
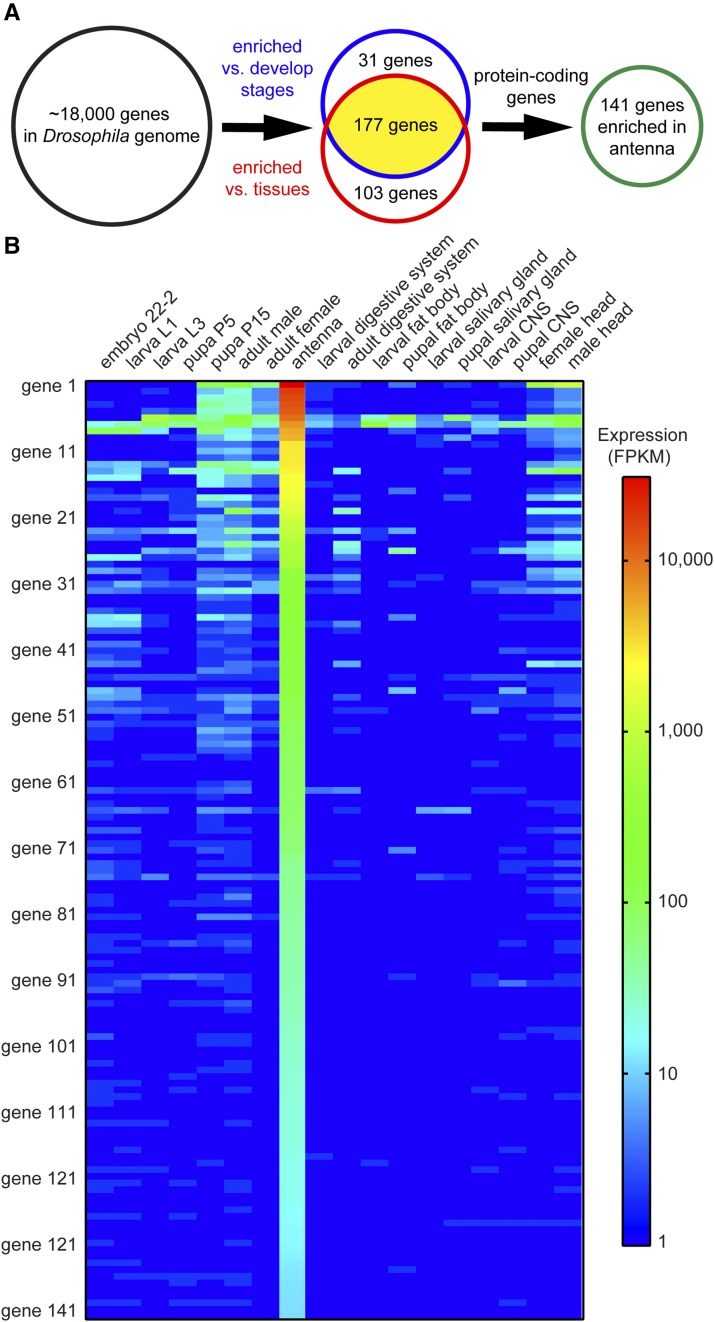
Computational identification of 141 antennal-enriched genes in *Drosophila*. (A) Summary of bioinformatic pipeline to identify antennal-enriched (AE) genes in *Drosophila*. Of >17,000 Drosophila genes, expression of 280 genes was >10-fold higher in antennae compared to any of ten tissue samples, and expression of 208 genes was >10-fold higher than seven samples of whole-bodies from different developmental stages. A subset of 177 genes was found in both groups, and among these were 141 protein-encoding genes. (B) Heat map of expression in FPKM of the 141 AE genes in the tissues and whole-body samples examined.

In order to compare ModEncode data with our antennal data, we re-quantified gene expression in our wild-type samples using a similar computational pipeline as the ModEncode datasets (File S1, “Cufflinks”) (Graveley *et al.* 2011; Brown *et al.* 2014). We then compared the mean expression of each gene expressed in the antenna with its expression in ten ModEncode tissue datasets including heads, digestive systems, fat bodies, etc. (Figure 3) (Brown *et al.* 2014). We specifically chose several tissues enriched with neurons to rule out genes involved in neuronal function rather than specific roles in olfaction. By comparing expression across datasets, we identified genes whose expression was at least 10-fold higher in antennae compared to all other tissues examined. Given that the ModEncode data has integer values (0, 1, 2 FPKM, etc.), we conservatively required that any identified antennal-enriched genes should also have greater than 10 FPKM expression in the antenna to ensure it was truly 10-fold enriched. With these strict criteria, we identified 280 genes more than 10 times as abundant in the antennae compared to other tissues (Figure 3).

The ModEncode consortium has also profiled gene expression across whole body extracts from multiple developmental stages in *Drosophila* (Graveley *et al.* 2011). Genes with specific olfactory functions should also be more than 10-fold enriched in antennae compared to expression across the body at any one developmental stage. Using the same methods as above, we compared antennal expression with expression in six developmental stages ranging from embryos to adults. We included both male and female adults, in case there is sexual dimorphism in gene expression. We identified 208 genes whose expression was 10-fold enriched in antennae compared to these developmental stages (Figure 3).

Finally, we merged the two gene lists to identify 177 genes that are enriched in the antenna compared to both the tissue and developmental datasets. Computational characterization of the genes using FlyBase indicated that 36 genes are non-protein coding. Further analysis focused on the remaining 141 “antennal-enriched” (AE) protein-coding genes (Figure 3B, Table 2, File S1, “antennal enriched genes”).

**Table 2:** Function and localization of 141 antennal-enriched (AE) genes

FBgn ID	Symbol	FPKM	*amos*-depleted	*ato*-depleted	Function
**CHEMOSENSORY RECEPTORS**					
FBgn0031634	Ir25a	49			IR chemosensory receptor
FBgn0051718	Ir31a	11		*^a^*	IR chemosensory receptor
FBgn0035604	Ir64a	17		X	IR chemosensory receptor
FBgn0036757	Ir75a	44		X	IR chemosensory receptor
FBgn0036829	Ir75d	30		X	IR chemosensory receptor
FBgn0036937	Ir76b	70		X	IR chemosensory receptor
FBgn0052704	Ir8a	25		X	IR chemosensory receptor
FBgn0030715	Or13a	13	X		OR chemosensory receptor
FBgn0062565	Or19b	16	X		OR chemosensory receptor
FBgn0026398	Or22a	36	X		OR chemosensory receptor
FBgn0026397	Or22b	25	X		OR chemosensory receptor
FBgn0026395	Or23a	11	X		OR chemosensory receptor
FBgn0028946	Or35a	18		X	OR chemosensory receptor
FBgn0033043	Or42b	76	X		OR chemosensory receptor
FBgn0026389	Or43a	21	X		OR chemosensory receptor
FBgn0026386	Or47a	45	X		OR chemosensory receptor
FBgn0026385	Or47b	86	X		OR chemosensory receptor
FBgn0034473	Or56a	21	X		OR chemosensory receptor
FBgn0034865	Or59b	82	X		OR chemosensory receptor
FBgn0041625	Or65a	28	X		OR chemosensory receptor
FBgn0041624	Or65b	32	X		OR chemosensory receptor
FBgn0041623	Or65c	10	X		OR chemosensory receptor
FBgn0036009	Or67a	14	X		OR chemosensory receptor
FBgn0036078	Or67c	13	X		OR chemosensory receptor
FBgn0036080	Or67d	41	X		OR chemosensory receptor
FBgn0041622	Or69a	14	X		OR chemosensory receptor
FBgn0030016	Or7a	23	X		OR chemosensory receptor
FBgn0037576	Or85a	17	X	X, *^b^*	OR chemosensory receptor
FBgn0037590	Or85b	21	X		OR chemosensory receptor
FBgn0038798	Or92a	85	X		OR chemosensory receptor
FBgn0039551	Or98a	39	X		OR chemosensory receptor
FBgn0030204	Or9a	35	X		OR chemosensory receptor
FBgn0037324	Orco	894	X		OR chemosensory receptor
FBgn0045502	Gr10a	23	*^c^*		GR chemosensory receptor
FBgn0041250	Gr21a	11	X		GR chemosensory receptor
FBgn0052255	Gr64f	31	X		GR chemosensory receptor
**ODORANT BINDING PROTEINS**					
FBgn0020277	lush	1,718	X		Odorant Binding Protein
FBgn0031109	Obp19a	2,997	X		Odorant Binding Protein
FBgn0011280	Obp19d	30,847			Odorant Binding Protein
FBgn0011283	Obp28a	5,444	X		Odorant Binding Protein
FBgn0034766	Obp59a	1,261		X	Odorant Binding Protein
FBgn0011279	Obp69a	3,026	X		Odorant Binding Protein
FBgn0011281	Obp83a	15,113	X		Odorant Binding Protein
FBgn0010403	Obp83b	17,591	X		Odorant Binding Protein
FBgn0011282	Obp84a	2,156		X	Odorant Binding Protein
**SMALL SECRETED PROTEINS, unknown functions**					
FBgn0010401	Os-C	12,694		X	
FBgn0011293	a10	13,648		X	
FBgn0259831	CG34309	190	X		
FBgn0259098	CG42246	46			
FBgn0040688	CG12483	96	X	X	
FBgn0261501	CG42649	39			
FBgn0262540	CG43094	140			
FBgn0262541	CG43095	11			
FBgn0085209	CG34180	40			
FBgn0263654	CG43645	88			
FBgn0034486	CG13869	15			
FBgn0262856	CG43220	604			
**JUVENILE HORMONE BINDING PROTEIN**					
FBgn0037288	CG14661	2,725			haemolymph juvenile hormone binding protein
FBgn0039298	to	1,554			haemolymph juvenile hormone binding protein
FBgn0038850	CG17279	145		X	haemolymph juvenile hormone binding protein
**CILIA-RELATED PROTEINS**					
FBgn0034446	Arl6	67			BBSome
FBgn0033578	BBS4	44			BBSome
FBgn0037280	BBS5	39			BBSome
FBgn0034622	BBS9	37			BBSome
FBgn0032119	CG3769	14			dynein
FBgn0033140	CG12836	19		X	dynein
FBgn0034481	CG11041	32		X	dynein regulatory complex
FBgn0050259	CG30259	67		X	dynein regulatory complex
FBgn0263076	Klp54D	18			kinesin
FBgn0050441	IFT20	35			intraflagellar transport
FBgn0032692	IFT46	20			intraflagellar transport
FBgn0031829	IFT52	31			intraflagellar transport
FBgn0038358	Ttc26	21			intraflagellar transport
FBgn0038814	CG15923	11			meckelin
FBgn0038170	CG14367	117			cilium assembly
FBgn0033685	OSCP1	97	X		cilium assembly
**DEGRADATION & METABOLISM**					
FBgn0026268	antdh	2,954			oxidoreductase
FBgn0034076	Jhedup	2,263	X		carboxylesterase
FBgn0051809	CG31809	79			oxidoreductase
FBgn0085371	CG34342	76	X		oxidoreductase
FBgn0263830	CG40486	994	X		oxidoreductase
FBgn0030949	Cyp308a1	178			cyp450
FBgn0000473	Cyp6a2	608	X		cyp450
FBgn0033696	Cyp6g2	115		X	cyp450
FBgn0033697	Cyp6t3	89			cyp450
FBgn0038029	GstD11	179	X		glutathione S transferase
FBgn0010044	GstD8	185	X		glutathione S transferase
FBgn0063496	GstE4	2,287	X		glutathione S transferase
FBgn0053177	CG33177	176	X		glutathione S transferase
FBgn0053178	CG33178	339			glutathione S transferase
FBgn0026314	Ugt35b	674			UDP-glucoronosyl transferase
FBgn0034605	CG15661	147			UDP-glucoronosyl transferase
FBgn0038350	AOX4	52		X	aldehyde oxidase
FBgn0038732	CG11391	1,072			acyl-coenzyme A (CoA) synthetase
FBgn0051216	Naam	134		X	nicotinamide amidase
FBgn0035453	CG10357	204		X	lipase
**KINASES**					
FBgn0032083	CG9541	96	X		kinase
FBgn0050274	CG30274	47	X		kinase
FBgn0031730	CG7236	66			kinase
FBgn0259712	CG42366	12			kinase
FBgn0031800	CG9497	2,031			kinase
**TRANSCRIPTION FACTORS**					
FBgn0034520	lms	12			transcription factor
FBgn0003513	ss	35			transcription factor
FBgn0052006	CG32006	38			transcription factor
**PEPTIDASE-RELATED PROTEINS**					
FBgn0052271	CG32271	94			peptidase
FBgn0053159	CG33159	79		X	peptidase
FBgn0040532	CG8369	6,966			proteinase inhibitor
**PROTEINS WITH OTHER FUNCTIONS**					
FBgn0259231	CCKLR-17D1	33			neuropeptide GPCR
FBgn0260004	Snmp1	294	X		CD36 lipoprotein receptor
FBgn0033110	CG9447	18			acyl transferase
FBgn0031254	CG13692	21			ARF/SAR small GTPase
FBgn0032181	CG13133	188		X	chaperone
FBgn0028658	Adat1	143			adenosine deaminase
FBgn0039864	CG11550	657			lipophilic ligand binding protein
FBgn0020660	eIF4B	14			translation initiation factor
FBgn0039386	CG5948	108			superoxide dismutase
FBgn0035434	Drsl5	10,249			anti-fungal peptide
FBgn0038309	Amt	209		X	ammonium transporter
FBgn0053349	ppk25	28	X	X	ppk sodium channel
FBgn0036235	CG6938	23	X		anoctomin family
**PROTEINS WITH UNKNOWN FUNCTIONS**					
FBgn0259179	CG42284	151			ankyrin repeats
FBgn0052668	CG32668	17			Arm repeat
FBgn0031289	CG13950	77		X	galectin domain
FBgn0037836	CG14692	20			IQ motif
FBgn0038397	CG10185	44		X	WD40 repeat
FBgn0039673	CG7568	25		X	WD40 repeat
FBgn0042129	OS9	5,104	X		
FBgn0011294	a5	2,995		X	
FBgn0033851	CG13332	15			
FBgn0035000	CG13578	152			
FBgn0036143	CG14142	92		X	
FBgn0038579	CG14313	26			
FBgn0263656	CG43647	23	X		
FBgn0039523	CG12885	22			
FBgn0032850	Kua	80	X		
FBgn0033629	Tsp47F	138			
FBgn0263655	CG43646	17			
FBgn0050285	CG30285	234			
FBgn0037014	CG13251	12			
FBgn0028740	CG6362	26			
FBgn0037492	CG10050	278		X	

a IR31a is known to be expressed in *ato*-dependent ac1 sensilla (Benton *et al.* 2009; Menuz *et al.* 2014). It nearly met the threshold for *ato*-depletion (3.9-fold reduced *vs.* 4-fold reduced).

b Or85a is known to be expressed in single-walled ab2 sensilla (Couto *et al.* 2005; Fishilevich and Vosshall 2005). Its reduction in *ato* flies is likely due to its location under the *Df(3R)p^13^* deficiency used with *ato^1^* (Menuz *et al.* 2014).

c Gr10a is known to be expressed in *amos*-sensitive ab1 sensilla (Fishilevich and Vosshall 2005). Due to overlapping annotation with Or10a, it was not detected by HTSeq.

### Validation of the bioinformatic identification approach

**Figure 4:**
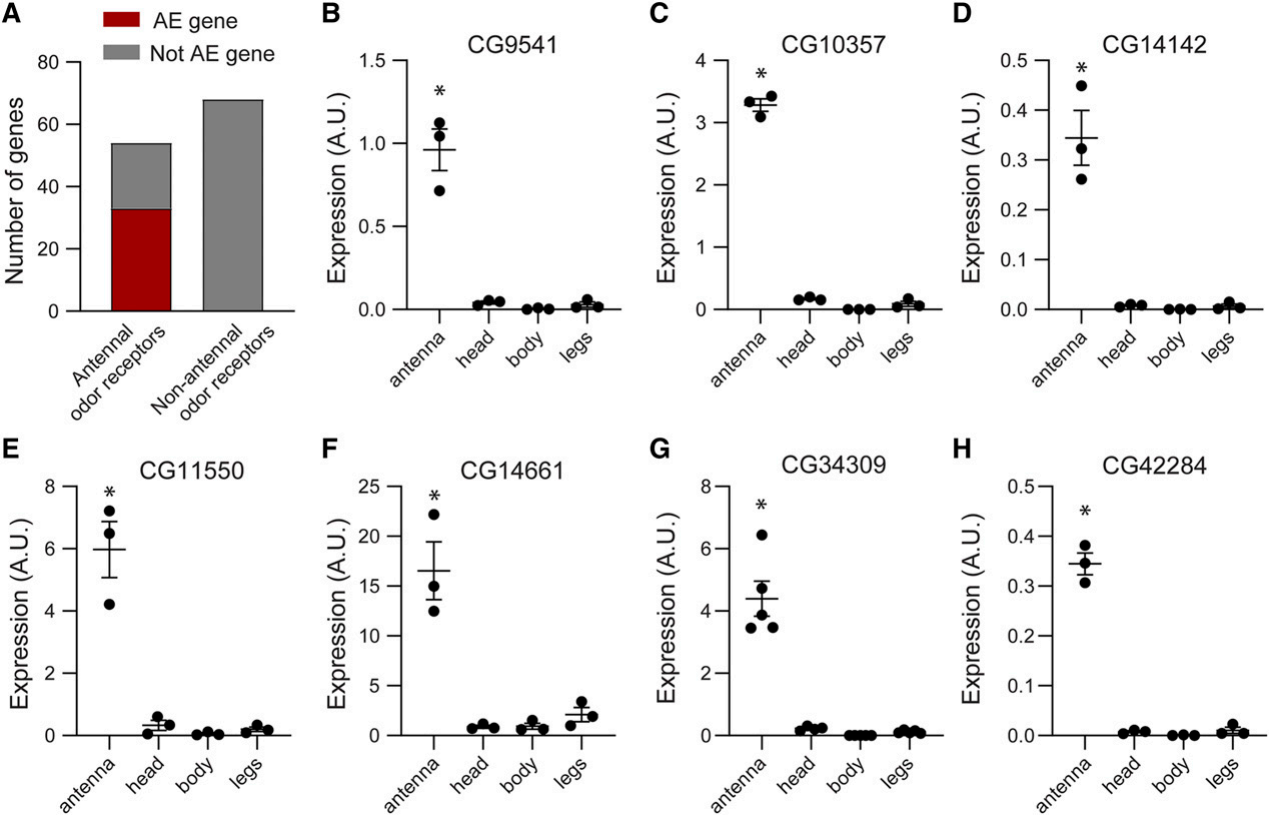
Validation of newly identified AE genes. (A) Graph of IR and OR family odor receptors, some of which are known to be expressed in the antenna and others that are not. The AE gene list includes ∼2/3 of known antennal odor receptors, whereas no non-antennal odor receptor is included. (B-H) Expression of seven AE genes was compared in antennae, heads, bodies and legs with qRT-PCR. For each, antennal expression is significantly higher than in the other tissues (n = 3-5, *P* < 0.001, one-way ANOVA with Dunnett’s *post hoc* test).

Our strategy relies on the ability to compare gene expression levels in our dataset to the ones generated by ModEncode previously. We therefore used two methods to validate our list of AE genes. First, we considered members of the OR and IR receptor families that are known to be expressed in the antenna (Figure 4A) (Couto *et al.* 2005; Fishilevich and Vosshall 2005; Silbering *et al.* 2011). Of these, ∼2/3 are contained within the set of AE genes. The other known antennal odor receptors generally had very low expression in the wild-type antennae, consistent with previous studies (Shiao *et al.* 2013; Menuz *et al.* 2014), and were therefore automatically excluded from the list of AE genes. We also looked at other OR and IR family members that are *not* expressed in the adult antenna, but rather in gustatory tissues or larvae (Couto *et al.* 2005; Sánchez-Alcañiz *et al.* 2018). As expected, none of these genes was identified as antennal-enriched.

As a second validation method, we used quantitative real-time PCR (qRT-PCR) to quantify the expression levels of antennal-enriched genes in tissues from wild-type flies. We investigated seven AE genes whose expression and function have never been directly examined. Each of these seven genes was detected at a significantly higher level in antennae compared to heads, legs, and bodies (*P* < 0.001, n = 3-5) (Figure 4B-H). Gene expression in each tissue for each gene was more than 10-fold lower than in antennae, with the exception of *CG14661* expression in legs. *CG14661* was detected at only ∼8 fold lower levels in legs than in antennae, perhaps due to the presence of leg gustatory sensilla which share many similarities to olfactory sensilla.

### Orthologs of AE genes have conserved antennal expression

We next sought to determine if these 141 AE genes found in *Drosophila* are also expressed in the antennae of distantly related insect species, which would be consistent with a conserved olfactory function. We chose to focus on the 96 genes that were not known odor receptors or OBPs, as the antennal expression of these gene families has been extensively studied in multiple insect species. We identified existing antennal RNA-Seq datasets on the NCBI Sequence Read Archive (SRA) from four insect species: mosquitoes (*Anopheles gambiae*, Diptera), bees (*Apis mellifera*, Hymenoptera), ants (*Harpegnathos saltator*, Hymenoptera), and beetles (*Tribolium castaneum*, Coleoptera). These are a diverse set of insects with a nearest common ancestor over 300 million years ago (Missbach *et al.* 2014).

**Figure 5:**
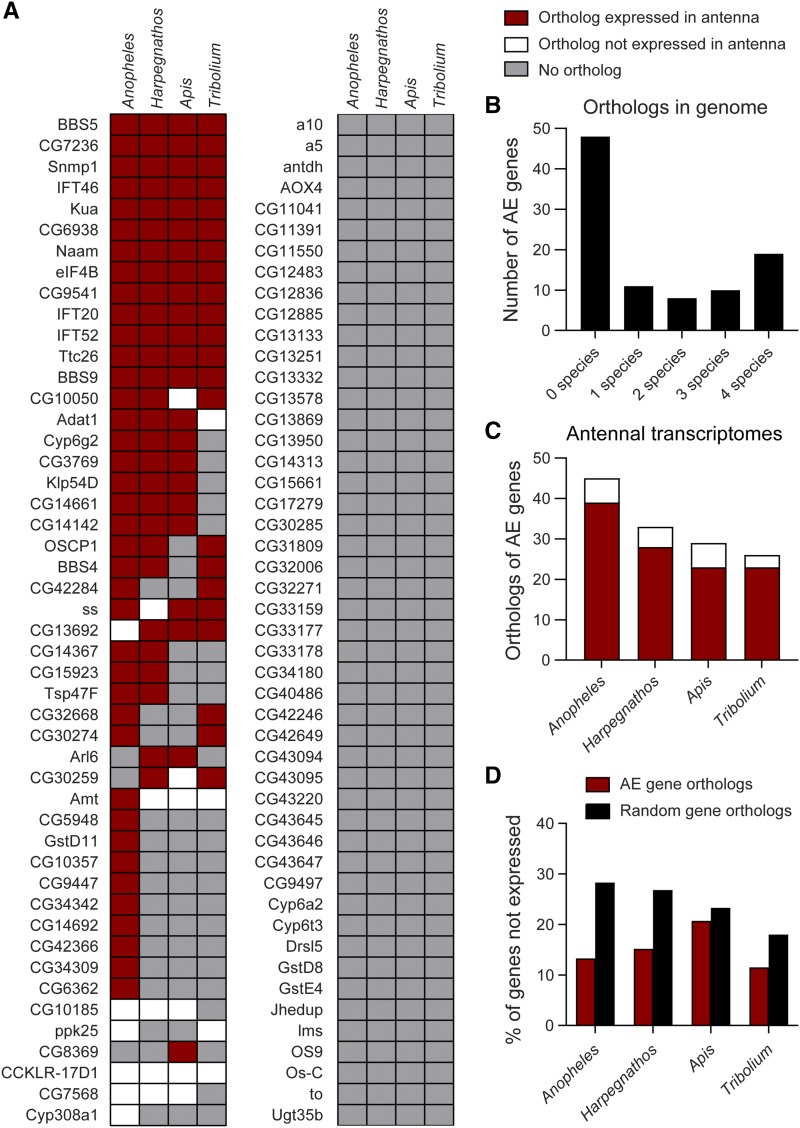
Orthologs of AE genes are expressed in the antennae of other insect species. (A) At least one of the four other insect genomes examined (*Anopheles*, *Harpegnathos*, *Apis*, *Tribolium*) contained an unambiguous ortholog for 48 of the 96 AE genes examined. The box shading indicates if a genomic ortholog of the AE gene was identified (white or red) or not (gray) in a particular species. Red signifies that the ortholog is expressed in the antenna of that species, whereas white indicates the ortholog is not expressed. (B) The graph depicts the number of AE genes with genomic orthologs found in 0, 1, 2, 3 or 4 of the examined species. (C) The graph shows the number of genomic orthologs of AE genes found in each insect species and the proportion of those orthologs that were detected in the antennal transcriptome of that species. Similar coloring as in A. (D) The graph shows the percentage of the AE gene orthologs and orthologs of randomly selected genes not expressed in each species.

We used the InParanoid 8 program to identify unambiguous orthologs of the 96 AE genes in the proteome of each of the four target insect species (Sonnhammer and Östlund 2014). With this program, we found an ortholog in at least one of the four insect species for half of the 96 AE genes, with 18 genes having orthologs in all four species (Figure 5A, B). Other genes either had no orthologs or had gene duplication events (paralogs), making it unclear which would be the functional ortholog of the *Drosophila* AE gene. The species with the most identified orthologs was the *Anopheles* mosquito, the species most closely related to *Drosophila* flies (Figure 5C).

We then used the coding sequences of these orthologous genes to interrogate the antennal transcriptomes of the relevant species using NCBI SRA-BLAST. In each of the four species, 79–89% of the orthologs were antennal-expressed (Figure 5A, C). Although this fraction is higher than the fraction of genes expressed in a typical tissue (60–70%) (Ramsköld *et al.* 2009), it is possible that highly conserved genes are particularly likely to be expressed. To test this possibility, we applied the same analysis to randomly selected *Drosophila* genes (Figure S1A). In each transcriptome analyzed, a greater fraction of the orthologs of random genes were not expressed than the fraction of AE genes (Figure 5D). If we raised the threshold for defining antennal expression to become more stringent, the difference between AE and random orthologs grew even larger (Figure S1B).

Surprisingly few random genes were not expressed in *Tribolium* (18%), and we wondered if this might reflect contamination of the sample, and thereby bias our interpretation of the AE gene expression. We therefore identified a second *Tribolium* antennal dataset with a smaller fraction of randomly selected genes expressed. Again, the AE genes were more often expressed than the randomly selected genes (Figure S1C). We note that the fraction of AE orthologs expressed in the *Apis* transcriptome was only slightly more than the fraction of expressed random orthologs, unlike the other three species. We therefore expanded our analysis to a fifth species, *Bombyx mori*. We found that the fraction of expressed AE orthologs was much greater than that of the randomly selected genes, similar to *Anopheles*, *Harpegnathos*, and *Tribolium* (Figure S1D). Together, our data support the possibility that more AE gene orthologs are expressed in the antennal transcriptomes than would be expected by chance alone.

### Differential localization of antennal-enriched genes

**Figure 6:**
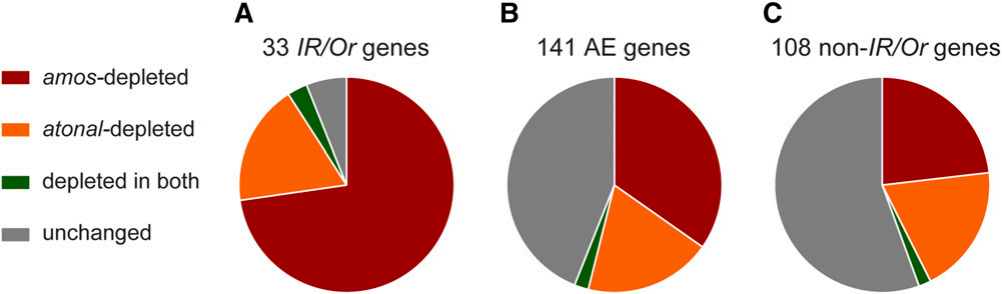
Localization of AE genes to morphological subtypes of sensilla. (A) The majority of IR and OR odor receptors could be localized to either single or double-walled sensilla based on their depletion in either *amos* or *atonal* antennae. (B) Over half of the 141 AE genes are depleted in either *amos* or *atonal* flies. A few genes were depleted in both genotypes (green) and half were unchanged in both mutants (gray). (C) Nearly half of the 108 non-chemoreceptor AE genes could be localized to a sensilla class.

Having identified 141 AE genes in *Drosophila*, we wondered whether some of the genes might function within signaling pathways specific to single or double-walled sensilla, similar to the odor receptor families themselves. Our first dataset revealed genes that are depleted in *amos* mutants and are therefore likely to be enriched in single-walled sensilla. We had previously generated a similar dataset for *atonal* flies, which selectively lack double-walled sensilla (Jarman *et al.* 1994; Menuz *et al.* 2014). We therefore inferred the localization of the 141 AE genes by cross-referencing the *amos*-depleted and *atonal*-depleted gene sets. The accuracy of this localization was examined by considering the 33 odor receptor AE genes. Nearly all of the OR and IR genes were depleted in either *amos* or *atonal* antennae in a manner consistent with their known localization (Figure 6A).

Including the odor receptors, we found that 49 of the AE genes were *amos*-depleted and 27 were *atonal*-depleted (Figure 6B, Table 2). Considered alone, nearly half of the 108 non-odor receptor AE genes could be localized to one of the two morphological sensilla classes, with a quarter of the genes depleted in either *amos* or *atonal* antennae (Figure 6B, Table 2). Further confirming our genetic localization strategy, we correctly mapped seven *amos*-depleted OBPs that had been previously detected in single-walled sensilla and two *atonal*-depleted OBPs that were previously found in double-walled sensilla (Table 2) (Larter *et al.* 2016). *Obp19a*, the only OBP AE gene not depleted in *amos* or *atonal* antennae, was previously shown to be expressed in epithelial cells that are not specific to either sensilla class (Larter *et al.* 2016). We expect that some other “non-localized” AE genes also play a role in cells outside of sensilla; another possibility is that some contribute to the function of both single and double-walled sensilla.

### Molecular functions of antennal-enriched genes

We next examined the putative molecular functions and predicted subcellular localization of the 141 AE genes (Table 2). We did this using web-based tools including DAVID, FlyBase, and DeepLoc (Huang *et al.* 2009; Almagro Armenteros *et al.* 2017; Thurmond *et al.* 2019). For some, we also ran BLAST searches to identify similar genes with known functions in other species.

The 141 AE genes include many known antennal chemosensory receptors, which represent 25% of the AE genes. Interestingly, 17% of the AE genes are small, secreted proteins with poorly characterized functions. This includes 9 OBPs, 3 members of the haemolymph juvenile hormone-binding protein family, and 12 additional proteins of unknown function. Of these, the OBPs have been best studied. Their exact function is unclear, but they have been hypothesized to be involved in solubilizing hydrophobic odorants, protecting odorants from degradation, odorant buffering, or delivery of odorants to receptors (Leal 2013; Larter *et al.* 2016). It is possible that the newly identified small, secreted proteins have similar roles. One difference is that nearly all OBPs were enriched in either single- or double-walled sensilla, whereas nearly all of the other small, secreted proteins distribute more broadly.

Insect antennae are densely covered with olfactory sensilla, and these sensilla have internal structures resembling cilia (Keil 1999; Keil 2012). It is therefore unsurprising that 16 (11%) of the AE genes are related to ciliary function, such as components of the BBSome and intraflagellar transport complexes (Table 2). Three genes related to the motor dynein are *atonal*-depleted genes, indicating that cytoskeletal transport may differ in the two sensilla classes.

Genes that may be related to odorant degradation comprise another 14% of the AE genes. A few of these genes, including *antdh*, *Jhedup*, and *Cyp308a1*, have been previously identified as antennal enriched (Wang *et al.* 1999; Younus *et al.* 2014; Steiner *et al.* 2017). More than half of these genes are localized to either single- or double-walled sensilla.

Smaller numbers of AE genes have more specialized functions. For example, three are transcription factors and three are related to peptidases. Other predicted functions include kinases, ion channels, transporters, and receptors. There are also a large number (15%) of AE genes with no predicted function.

## Discussion

We have generated two resources for studying the molecular basis of insect olfaction beyond the function of the odor receptors themselves. We identified 187 genes enriched in single-walled sensilla that could contribute to the detection of food odors, pheromones, and attractive human-derived odors. Most of these genes are previously unstudied in the antenna, and their predicted functions of many suggest they could have roles in regulating olfactory neuron signaling. We also describe 141 antennal-enriched genes, of which some including *lush*, *Snmp1*, *Amt*, *spineless* and OR/IR odor receptors have established olfactory roles (Dong *et al.* 2002; Xu *et al.* 2005; Benton *et al.* 2007; Menuz *et al.* 2014). Thus, we expect many of the other AE genes will also contribute to olfaction in *Drosophila*. Excitingly, most of the identified orthologs of AE genes were expressed in the antennae of distantly related insect species, suggesting that many may have evolutionarily conserved roles.

Our study of *amos*-depleted genes unexpectedly identified a number of non-protein coding RNAs that are differentially expressed in single and double-walled sensilla. Several are antisense RNAs that target chemosensory receptors (*Or46a*, *Or88a*, *Gr64d*, and *Gr64e*). In other systems, antisense RNAs are increasingly reported to regulate gene expression (Kashi *et al.* 2016), but to our knowledge a role for antisense RNAs in olfactory physiology has not been studied. Such a regulatory system could underlie changes in odor receptor activity in response to circadian rhythms and prolonged odor exposure (Krishnan *et al.* 1999; von der Weid *et al.* 2015).

Recent reports indicate that several members of the gustatory receptor family are unexpectedly found in the antenna (Menuz *et al.* 2014; Fujii *et al.* 2015). The localization and function of most antennal GRs is unknown. Our data from *amos* flies indicate that many GRs are found within single-walled sensilla. It will be interesting to determine if they are co-expressed with OR receptors within ORNs, such as *Or10a* and *Gr10a (**Fishilevich and Vosshall 2005**)*, or if there are unidentified ORNs that exclusively use these receptors such as those utilizing *Gr21a*/*Gr63a* (Jones *et al.* 2007). There is at least one antennal lobe glomerulus (VA7m) that is not mapped to a chemoreceptor (Grabe *et al.* 2016); perhaps a GR-expressing ORN projects to this glomerulus.

We observed that many of the genes enriched in single-walled sensilla could comprise distinct molecular signaling pathways that underlie their unique olfactory functions. These include seven GPCRs that could serve to differentially regulate the activity of ORNs in response to neuromodulators or hormones (Wilson 2013; Gadenne *et al.* 2016). The discovery of metabotropic serotonin and acetylcholine receptors enriched in single-walled sensilla suggests that these neuromodulators could act on ORN terminals in the antennal lobe similar to the previously described metabotropic GABA_B_ receptors (Olsen and Wilson 2008). Other *amos*-depleted GPCRs such as *hec*, implicated in mating behavior, and *Lgr1*, responsive to hormones, could affect the known activation of some single-walled sensilla ORNs to mating pheromones (Hauser *et al.* 1997; Li *et al.* 2011). Our dataset also identified signaling molecules, such as the G-protein * Gαf*, the kinases *CG30274* and *CG9541*, and the phospholipase A2 *CG42237*, which could be components of intracellular signal transduction cascades. Five members of the pickpocket family of ligand-gated cation channels are also *amos*-depleted. Although the functions of most family members are poorly understood, some are gated by neuropeptides and other small molecules and could act as neuromodulators (Zelle *et al.* 2013). Based on the enrichment of particular receptors and signaling molecules in single-walled sensilla, we speculate that neuromodulators differentially regulate the activity of ORNs in different morphological classes of sensilla, an area ripe for future investigations.

We found that a large number of AE genes encode enzymes with potential roles in odor degradation and that these are often depleted in either *atonal* or *amos* antennae. Additionally, there are a large number of such enzymes that are either *amos*-depleted or *atonal*-depleted, even if they are not antennal enriched. Together this indicates that different sensilla classes may utilize different degradation enzymes. One reason this might occur is sensilla may selectively express enzymes needed to metabolize odors their ORNs detect, with the segregation of enzymes for alcohols, esters, and long-chain hydrocarbon pheromones to single-walled sensilla and enzymes for amines and acids to double-walled sensilla (Steinbrecht 1997; Hallem and Carlson 2006; Silbering *et al.* 2011). An alternative possibility is that odors only accumulate in the peri-neuronal sensillar lymph in particular sensillar classes, perhaps due to restricted access by pores on the sensillar shaft (Steinbrecht 1997). If so, then all sensilla with similar pores, such as single- or double-walled sensilla, might need similar enzymes to prevent odor accumulation.

The structure of insect ORN outer dendrites is thought to resemble a modified cilium (Keil 1999; Keil 2012), but only limited work has characterized the effect of ciliary mutants on olfactory function. We identified 16 AE genes involved in ciliary function, including four members of the basal body complex (BBSome) and four involved in intraflagellar transport that both contribute to the assembly and maintenance of cilia (Avidor-Reiss *et al.* 2004; Jana *et al.* 2016). Only a few ciliary genes are differentially expressed in *amos* and *atonal* mutants, suggesting similar ciliary structures are found in both single- and double-walled sensilla. Further, antennal expression of ciliary gene orthologs is highly conserved in the four insect species examined.

Although our approach sought to identify genes with exclusive roles in olfaction, it is clear that ciliary genes also contribute to the function of other ciliated cells and biological processes. Ciliary genes were most likely detected as antennal-enriched due to the dense tiling of olfactory sensilla on the antennal surface (Shanbhag *et al.* 1999), making the density of ciliated cells in this tissue far greater than in other tissues examined. More broadly, this also indicates that some AE genes may play a role in both the antennae and in other tissues.

Our study identified a large number of known olfactory genes and many more novel genes with potential roles in olfaction, but it is likely that some genes with relevance for olfaction were overlooked. By design, our computational approach discarded many genes that are important for olfaction and contribute to the physiology of other tissues. This may be the reason why we did not detect genes that are likely to maintain the transepithelial potential, an electrical potential that drives odor receptor transduction and likely relies on potassium ion movement (Kuppers and Thurm 1979; Stengl *et al.* 1999). Our approach also discarded genes with low abundance in the antenna to ensure that genes were truly antennal enriched. This conservative approached prevented some known antennal odor receptors from being included among the AE genes. Our strategy pooled approximately equal numbers of male and female antennae, preventing additional consideration of sexual dimorphism in gene expression, such as has been observed previously (Zhou *et al.* 2009). It will be interesting to determine if any of the identified genes are highly enriched in either males or females.

A key advantage of our approach is the identification of genes with potentially conserved roles in olfactory signaling. We discovered many AE genes with orthologs expressed in the antennae of all insects we examined, making them prime targets for future mechanistic investigations. When combined with information from our *amos*-depleted and *atonal*-depleted datasets, our identification of AE genes reveals new avenues for examining the mechanistic underpinnings of poorly understood processes that support faithful odor detection in the antenna.
